# Pancreatic cancer microenvironment, to target or not to target?

**DOI:** 10.15252/emmm.201505948

**Published:** 2016-01-08

**Authors:** Ryan M Carr, Martin E Fernandez‐Zapico

**Affiliations:** ^1^ Department of Internal MedicineMayo ClinicRochesterMNUSA; ^2^ Schulze Center for Novel TherapeuticsMayo ClinicRochesterMNUSA

**Keywords:** Cancer, Digestive System

## Abstract

We have collectively been spoiled by the astounding clinical benefit of antimicrobials. Much like the discovery and use of penicillin to eradicate once deadly infections, we continue to desperately search for the next “magic bullet” to kill cancer while sparing the non‐transformed cells. Greater appreciation for the molecular intricacies of malignancy has resulted in dedicated pursuit of cancer genomics and large‐scale informatics to identify “drugable” targets within the cancer cell itself. However, studies at the bench elucidating a dynamic relationship between tumor and microenvironment have become more common and demonstrate promise for novel therapeutic intervention.

Cancer cells are known to stimulate the surrounding stromal cells while reciprocal signals are released to the tumor cells to promote their growth and invasion. The specific example of pancreatic ductal adenocarcinoma (PDAC) illustrates both the shortcomings of conventional therapies and the importance of the PDAC microenvironment in clinical outcomes.

PDAC is a highly lethal malignancy with ~80% of patients presenting with locally advanced disease. These patients are typically not eligible for surgical intervention and desperately need effective medical therapy. This malignancy is largely resistant to both chemotherapy and radiation with < 6% 5‐year survival. Why this malignancy remains refractory to most therapies is still unresolved. There has been an increased interest in the potential targeting of the PDAC desmoplastic reaction, a cellular compartment containing cancer‐associated fibroblasts (CAFs), extracellular matrix proteins, inflammatory, and endothelial cells (Fig 1). Multiple strategies have been investigated, most prominently including depletion of cellular elements of desmoplasia, enhancing tumor perfusion through alleviation of intratumoral pressures, and local immunomodulation. However, the role of these strategies in treatment remains largely contentious.

**Figure 1:**
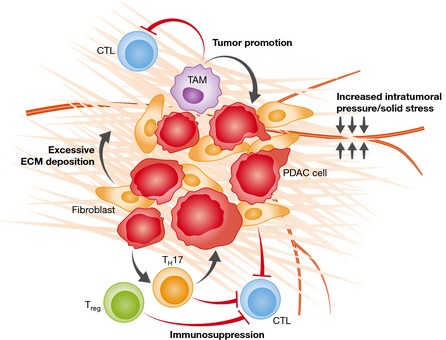
Interplay among the components of PDAC desmoplastic reaction Multiple key components of the PDAC microenvironment modulate the biology of PDAC. Cancer‐associated fibroblasts within the PDAC microenvironment are involved in deposition of the dense extracellular matrix (ECM) typical of the desmoplastic reaction. Dense ECM components confer elevated intratumoral pressures and solid stress resulting in vascular compression and reduced diffusion into the tumor interstitium. An immunosuppressive inflammatory infiltrate consisting of regulatory tumor‐associated macrophages (TAMs), T cells (Treg), and T_H_17 cells is recruited to the PDAC microenvironment. These cells play a key role in tumor promotion and dampening of cytotoxic T lymphocyte (CTL) response to the tumor.

## Modulating the desmoplastic reaction

Depletion of the tumor–stroma is currently a controversial strategy for PDAC treatment. Olive *et al* (2009) first demonstrated the potential benefit of Sonic hedgehog (Shh) inhibition in disrupting desmoplasia. Based on data from a genetically engineered mouse model (GEMM), Shh pathway inhibitor (IPI‐926) treatment yielded reduced tumor–stroma and increased survival compared to controls, while also increasing tumor vascularity and gemcitabine delivery. Consequently, it was hypothesized that the stroma confers chemoresistance to PDAC at least partly through decreased drug penetrance. Infinity Pharmaceuticals eventually started phase I and II trials with IPI‐926, but these were stopped due to poor clinical performance (http://phx.corporate-ir.net/phoenix.zhtml?c=121941&p=irol-newsArticle&ID=1653550&highlight=).

More recent contradictory findings have raised significant skepticism regarding stromal depletion as treatment. Rhim *et al* (2014) found that GEMM in which conditional Shh ablation resulted in diminished stroma formation, featured reduced survival due to formation of more aggressive, dedifferentiated tumors, and increased metastases. Long‐term administration of IPI‐926 was phenotypically similar possibly explaining the drug's failure in clinical trials. In parallel, Ozdemir *et al* limited the development of desmoplasia with conditional depletion of αSMA^+^ myofibroblasts. Once again, stromal depletion resulted in decreased survival with similarly aggressive tumors (Ozdemir *et al*, 2014). Rhim *et al* (2014) showed increased tumor vascularity with stromal depletion, which correlated with disease progression but also increased responsiveness to anti‐angiogenic agents.

Taken together, it could be concluded the PDAC desmoplasia actually plays a protective role for the host, making its targeting a much less appealing therapeutic strategy. However, the disparities between these preclinical models should trigger reassessment based on the relative importance of the stromal component being targeted and timing of the intervention. Interestingly, one common feature between the studies was increased tumor vascularity as a consequence of stromal depletion. Should we focus on the pharmacologic promotion of desmoplasia or can more specific targeting of stromal depletion be achieved, thus enhancing drug delivery while preventing increased aggressiveness? A more precise modulation of desmoplasia as opposed to global stromal depletion clearly deserves further investigation.

## Altering tumor vascularity

The PDAC microenvironment is hypoxic with minimal vascularity. Despite this relatively diminished vascularity compared to other tumor types, elevated pro‐angiogenic vascular endothelial growth factor A (VEGF‐A) levels in patients have been found to correlate with increased vascular density of PDAC and greater disease progression. PDAC treatment with anti‐VEGF‐A therapies such as bevacizumab and axitinib was therefore evaluated in clinical trials though their combination with gemcitabine fell short of improving survival. Thus, failure of anti‐angiogenic agents in clinical trials was likely due to limited penetrance. A theoretical therapeutic window for such targeted strategies might therefore be developed.

The dense extracellular matrix of PDAC confers remarkable biophysical rigidity with increased intratumoral pressures unparalleled by other malignancies. Increased pressure causes collapse of the vasculature and diminished diffusion into the tumor interstitium. This is hypothesized to be a major barrier to response to therapies. Can we develop therapeutic targets or strategies to alleviate these pressures and increase responsiveness to conventional therapy? Provenzano *et al* proposed increased interstitial pressures mediate blood vessel collapse, while others (Chauhan *et al*, 2014) suggest that vascular compromise is secondary to increased solid stress. Provenzano *et al* identified increased production of hyaluronic acids as the primary determinant of elevated intratumoral pressures. Indeed, treatment with human recombinant hyaluronidase (PEGPH20) relieved intratumoral pressures, increase in tumor vascular perfusion and gemcitabine delivery, improved survival, and decreased the metastatic burden (Provenzano *et al*, 2012; Jacobetz *et al*, 2013). However, a phase II clinical trial was prematurely stopped because of increased thromboembolic event risk in patients receiving PEGPH20. Because interstitial pressure is balanced by intravascular pressure, interstitial pressures cannot be persistently elevated to the point of vessel compression. Solid stress, capable of overcoming intravascular pressures, is mediated by extracellular matrix components (Chauhan *et al*, 2014). This distinction is critical as the tumor elements responsible for each stress are disparate. Thus, strategies aimed to mitigate solid stress in PDAC might yield therapeutic benefit.

## Immunological sensitization

An extensive stromal immunosuppressive inflammatory infiltrate, including regulatory T (T_reg_) cells, myeloid‐derived suppressor cells (MDSCs), and macrophages, appears early in PDAC tumorigenesis. Recently, Zhang *et al* showed high levels of both T_reg_ cells and T helper 17 (T_H_17) with few CD8^+^ lymphocytes in the microenvironment of the KRAS PDAC model. Depletion of the CD4^+^ T‐cell population resulted in derepression of anti‐tumoral CD8^+^ T lymphocytes with prevention of tumor progression (Zhang *et al*, 2014). McAllister *et al* (2014) described a “IL‐17 signaling axis” in which oncogenic Kras is important in recruitment of T_H_17 cells. IL‐17 signaling from T_H_17 cells is then integral in the formation of pre‐invasive lesions and promotion of PDAC. The tumor‐associated macrophages (TAMs) are also critical in mediating PDAC immune escape. Depletion of a specific extra‐tumoral macrophage population has been demonstrated to enhance CD8^+^ T‐cell tumor infiltration in response to CD40 agonist immunotherapy (Beatty *et al*, 2015). Conversely, activation of the stromal macrophages by BAG3–IFITM‐2 axis promotes tumor growth. Blockade of this cascade using an anti‐BAG3 antibody diminished primary tumor growth and metastasis (Rosati *et al*, 2015). In addition to immune escape, infiltrating macrophages contribute to tumor formation and maintenance through production of cytokines RANTES and TNF‐a, which mediate acinar‐to‐ductal metaplasia, secretion of IL‐6 promoting tumor progression through STAT3 signaling, and by overexpressing cytidine deaminase, which inactivates gemcitabine (Liu *et al*, 2013). These functions make TAMs an obvious therapeutic target with molecules, such as trabectedin, and show promise in enhancing immunotherapy for PDAC.

While an important role of immunosuppressive cells in the initiation and progression of PDAC has been elucidated, our knowledge of the interactions between stromal elements and the inflammatory infiltrate is incomplete. Interestingly, CAFs and myofibroblasts may partly mediate local immune suppression. Feig *et al* (2013) depleted fibroblast activation protein‐α‐expressing CAFs resulting in increased anti‐tumor cytotoxic T‐cell‐mediated control of PDAC. This was recapitulated by blocking the activity of CAF‐secreted cytokine CXCL12, preventing activity on tumor cells to effectively exclude anti‐tumor T cells. Abrogation of CXCL12 enhanced tumor response to immune checkpoint therapy, potentially explaining the known resistance of PDAC to therapies such as pembrolizumab (a PD‐1 antagonist). Similarly, αSMA^+^ myofibroblast elimination caused decreased CD4^+^ effector T cells, decreased cytotoxic T lymphocytes, and increased regulatory T cells but also mediated an increased susceptibility to checkpoint antagonists (Ozdemir *et al*, 2014). Thus, targeting of stromal elements opens a therapeutic opportunity to increase the efficacy of targeted therapeutics, including immunomodulatory approaches, thus far ineffective in PDAC treatment.

## Conclusion

Therapeutic advancement for PDAC has been negligible. Shortcomings are likely due to the cancer cell‐centric approaches to solid tumor therapeutics. Conventionally viewed as the result of random somatic mutations conferring unopposed proliferation, cancer must now be considered within the context of a biologic system, a tumor organ of sorts. Furthermore, as the extensive inter‐ and intratumoral heterogeneity is further characterized, so must the heterogeneity of tumor microenvironment. “Virtual microdissection” of PDAC gene expression data has not only allowed identification of tumor subtypes, but also two distinct stromal types (Moffitt *et al*, 2015). Stromal heterogeneity may at least partially explain the contradictory findings in preclinical models targeting different microenvironment elements of PDAC, although the relationship between GEMM phenotypes and their human counterparts is unclear. Nonetheless, increased complexity and heterogeneity suggests the need for personalized medicine to make therapeutic advances in PDAC and likely other cancers. With our exponentially increasing understanding of the molecular basis of PDAC and its microenvironment, this is an exciting time as we shift our focus to the tumor–stroma relationship to design new therapies for a currently deadly disease.
